# Bacteriology and Antibiogram of Urinary Tract Infection Among Female Patients in a Tertiary Health Facility in South Eastern Nigeria

**DOI:** 10.2174/1874285801711010292

**Published:** 2017-10-31

**Authors:** Angus N. Oli, Vivian B. Akabueze, Chijioke E. Ezeudu, George U. Eleje, Obiora S. Ejiofor, Ifeanyichukwu U. Ezebialu, Charlotte B. Oguejiofor, Ifeoma M. Ekejindu, George O. Emechebe, Kenneth N. Okeke

**Affiliations:** 1 Department of Pharmaceutical Microbiology and Biotechnology, Faculty of Pharmaceutical sciences Agulu, Nnamdi Azikiwe University, Awka, Nigeria; 2 Department of Peadiatrics, Faculty of Medicine, College of Health Sciences, Nnamdi Azikiwe University, Nnewi Campus, Anambra state, Nigeria; 3 Department of Obstetrics and Gynaecology, Faculty of Medicine, College of Health Sciences, Nnamdi Azikiwe University, Nnewi Campus, Anambra state, Nigeria; 4 Department of Peadiatrics, Chukwuemeka Odumegwu Ojukwu University, Awka Campus, Anambra state, Nigeria; 5 Department of Obstetrics and Gynaecology, Chukwuemeka Odumegwu Ojukwu University Awka Campus, Anambra state, Nigeria; 6 Department of Medical Laboratory Science, Faculty of Health Science and Technology, College of Science, Nnamdi Azikiwe University, Nnewi Campus, Anambra state, Nigeria

**Keywords:** *Bacteriology*, *Antibiogram*, *Urinary tract infection*, *Bacteriuria*, *Uropathogens*

## Abstract

**Background:**

Urinary Tract Infection (UTI) is a common contagion among men and women with the incidence relatively higher among women due to their differing anatomy. An understanding of the kind of pathogens implicated in urinary tract infections as well as antibiotic susceptibility profiling may help the clinician make rationally correct empirical choice in their treatment.

**Objective:**

This study is aimed at determining the type and antibiotic susceptibility pattern of bacterial uropathogens isolated from female patients attending Chukwuemeka Odumegwu Ojukwu University Teaching Hospital (COOUTH), Awka, Nigeria.

**Method:**

Two hundred and forty patients with clinically diagnosed UTI and who were on at least 5 days’ antibiotic holiday were recruited into the study. Their demographic characteristics were captured using pre-tested questionnaire. Their clean catch mid-stream urine samples were collected using sterile universal container and sent to the Microbiology Department for processing. Within 30 minutes of samples collection, the specimens were cultured and the isolates were identified, after 24 h of incubation, using standard microbiological techniques. Antibiotic susceptibility tests were done with standard antibiotic discs using the Kirby–bauer disc diffusion method.

**Results:**

Out of the 240 urine samples, 89.17% yielded significant bacteriuria. The pathogens implicated were *Escherichia coli* (28.5%), *Staphylococcus aureus* (28.0%), *Salmonella* spp (22.8%) and *Pseudomonas aeruginosa* (20.5%). HIV status, patients age, pregnancy status and marital status all significantly affected bacteriuria rate (p value < 0.05), while patients’ location (sub-urban/rural dwelling), and level of education did not (p value > 0.05). The pattern of microbial resistance to antibiotics suggests that ceftazidime, fosfomycin and cefoxitin may not be used as first-line agents in the empirical treatment of UTIs rather; levofloxacin, meropenem or aztreonam should be considered. Levofloxacin was significantly effective against all the isolates and may be administered empirically while waiting for the culture result (Mean % susceptibility was 79.85).

**Conclusion:**

*E. coli* and *S. aureus* were the predominant pathogens in the study and many were resistant to the commonly prescribed antibiotics and so leave the clinicians with only few alternative drugs for UTIs treatment. Routine surveillance and monitoring studies need to be constantly conducted to update clinicians on the prevalent pathogens and the rational and empirical treatment of UTIs. Aggressive and consistent health education using every possible media is also recommended to combat the menace of drug resistance occasioned by inappropriate antibiotic use.

## 1 INTRODUCTION

Urinary Tract Infections (UTIs) have been proven to be the most encountered bacterial infection in humans [1, 2], affecting all age groups and gender in both the community and hospital settings [3, 4]. About 50% of all females will experience at least an episode of UTI during their lifetime [5]. Asymptomatic bacteriuria and urinary tract infection (UTI) are common among adult men and women; although the incidence is higher among women due to their anatomy [6, 7]. They also have a higher prevalence among women during pregnancy and in very sexually active females [8]. Asymptomatic bacteriuria denotes significant bacteriuria (> 10^5^ CFU/mL of urine) without clinical symptoms of urinary tract infections (such as frequent urination, painful urination or fever) or other abnormal findings. The bacteriuria should not be due to contamination from urine sample collection.

UTI is an infection that affects any part of the urinary tract from the bladder to the kidney. It is not classified as a sexually transmitted infection [9] although sexual activity is known to be a risk factor [10]. Symptoms include frequent and/or painful urination, a feeling to urinate despite having an empty bladder, fever and flank pain. At times, the urine may contain pus and/or appear bloody. UTI is a risk factor for pyelonephritis, preterm delivery and miscarriages among pregnant women, and is associated with impaired renal function and end-stage renal disease among pediatric patients [11].

Antibiotic resistance in the treatment of UTI and other bacterial infections constitute a major public health problem especially in the developing countries. Irrational and indiscriminate use of antibiotics as well as fake and substandard drugs, including antibiotics is common in these countries [12, 13]. In view of these and attendant tendency for changes in bacteriological profile, it is worthwhile that the degree of susceptibility and resistance of these uropathogens to various antibiotics be known to clinicians for effective treatment of infections they cause and to avoid antibiotic miss-use. This study is aimed at determining the type and level of drug susceptibility of bacterial uropathogens isolated from female patients with symptomatic UTI and attending a tertiary health care institution in south-east Nigeria. This will help reduce irrational use of antibiotics and development of resistance.

## 2 MATERIALS AND METHODS

### 2.1 Study Area and Study Population

This study was carried out at Chukwuemeka Odumegwu Ojukwu University Teaching Hospital (COOUTH), Awka, Nigeria; an Anambra State owned 150 bedded Hospital serving over 2 million populations. The study population comprised symptomatic female patient aged 12-56 years who presented at the peadiatric, obstetrics and gynaecology and antenatal out-patient clinics of the hospital between May 1^st^ and July 31^st^ 2016. Participants were recruited consecutively after their verbal informed consents were obtained or that of their legal care-givers.

All the media used in the study were products of HiMedia Laboratories Pvt. Ltd, Mumbai, India while the antibiotic discs were from Oxoid, England.

### 2.2 Sample Size and Sample Technique

The minimum sampling size was determined using the statistical formula:

N = (Z^2^pq)/ D^2^ where

q = (1-p)

N = Sample Size

p = Prevalence Rate in %

Z= Confidence interval of 95% which is equivalent to Confidence of 1.96

D = Desired level of Size Significance (5%)

The prevalence of UTI in a similar hospital in South-east Nigeria was reported as 2.0% [14]

So, N = 30.12. Four study groups (Pregnant HIV positive, Pregnant HIV negative, Non- pregnant HIV positive and Non- pregnant HIV negative) were used with each group having a population of 60 subjects. A total sample size of 240 was chosen.

### 2.3 Study Design and Sample Collection

This is a cross-sectional hospital based study. Patients with clinical manifestations of UTI who gave consent were included in the study, while those with clinical manifestations but are on antibiotics within the past five days were excluded.

Clean catch mid-stream urine was collected, from each consenting patient in a 20 mL calibrated sterile screw-capped universal containers which were distributed to the patients. Each specimen was appropriately labeled, transported to the Microbiology Laboratory of the Department of Pharmaceutical Microbiology and Biotechnology, Faculty of Pharmaceutical Sciences, Nnamdi Azikiwe University, Agulu, Nigeria and analyzed within 30 minutes of collection. Prior to sample collection, all patients or their legal care-givers were well instructed by the recruiting peadiatrician and gynaecologist on how to collect clean catch mid-stream urine. Urine samples were collected from a total of 240 pregnant and non-pregnant females.

### 2.4 Sample Processing and Culture

On arrival at the laboratory, MacConkey agar, Mannitol salt agar, Cetrimide agar and Salmonella-Shigella agar were prepared according to their manufacturers’ specifications and sterilized in the autoclave by heating at 121°C for 15 minutes. The sterilized media were poured aseptically into previously labeled sterile petri-dishes and allowed to solidify. Each urine sample was aseptically inoculated (in triplicate) onto the MacConkey agar plates, Mannitol salt agar plates, Salmonella-Shigella agar and Cetrimide agar plates. The inoculated agar plates were incubated at 37°C for 24 h.

### 2.5 Identification of Isolates and Antibiogram Study

Identification of bacterial isolates was done on the basis of their cultural, Gram and biochemical characteristics while the antibiotic susceptibility of pure cultures of confirmed isolates was performed on Mueller-Hinton Agar using the standard Kirby-Bauer disc diffusion method. Briefly, standardized overnight culture of each isolate was prepared by inoculating 3-5 pure colonies of each isolate into 3 ml sterile nutrient broths in test tubes and adjusting to match 0.5 McFarland turbidity standards. The microbial suspensions were streaked on to the Mueller-Hinton Agar plates using a sterile wire loop and allowed to dry and diffuse for about 5 minutes. The antibiotic discs (Ceftazidime 30µg, Cefuroxime 30µg, Meropenem 10ug, Fosfomycin 50ug, Cefoxitin 30ug, Azithromycin 15ug, Levofloxacin 5ug, Ceftriaxone/Sulbactam (30/15ug), Aztreonam 30ug, Ceftriaxone 30ug.) were aseptically picked and placed on to the surface of the microorganisms seeded agar plates. The plates were incubated for 24 h at 37 ºC. The inhibition zones created by each antibiotic against each isolate were measured and recorded in millimeter (IZD in mm) as Inhibition Zone Diameter. Susceptibility/resistance was interpreted according to Clinical and Laboratory Standard Institute (CLSI) guidelines [15].

### 2.6 Data Analysis and Interpretation

The data were analysed using GraphPad Prism version 5.00 for Windows, GraphPad Software, Inc. San Diego California USA, www.graphpad.com. One-Way Analysis of Variance (ANOVA) was used to establish the mean differences in prevalence of the isolates among various age groups while the Chi Square checked for the relative risks of UTI in relation to pregnancy status, HIV status, marital status, educational levels and urban/rural dwellers. Two-Way Analysis of Variance (ANOVA) was used to establish the mean differences in susceptibility of the isolates to the commercial antibiotics. Bonferroni post-tests were used to compare the effects of the individual drugs on the isolates. All P values reported are for a two-tailed test. The significance level was chosen at p< 0.05.

### 2.7 Ethical Approval and Compliance with Ethical Standards

Ethical approval for the study was obtained from the ethical committee of COOUTH, Awka with reference number COOUTH/AA/VOL.1.006. All necessary international, national, and/or institutional ethical guideline were followed and the study protocols obeyed the 1964 Helsinki declaration and its later amendments or comparable ethical standards.

The participants or their legal care-givers gave a verbal consent to participate in the study after the reasons for the study were explained to them. No information on the participants’ names was collected. The urine sample containers and questionnaires for data collection were identified with study identification numbers.

## 3 RESULTS

Table **1** shows that at the time of the study, most of the UTI patients (47.92%) encountered were from rural communities. However, correlation analysis did not show that rural dwelling significantly correlate (p > 0.05) with the incidence of bacterial UTI. Age, marital status and educational levels also did not correlate (p > 0.05). There were significant differences in the incidence of bacterial UTI among the age groups and in the marital status of the study participants (p < 0.05).

Also, it can be observed that the incidence of UTI increased from urban to rural dwelling and also increased with increasing age of the participants until it picked at age 27-31. It was observed that the incidence of UTI decreased with increase in the level of formal education acquired, although this was not significant (p > 0.05). Marital Status significantly affected outcome (bacteriuria) – may be due to sexual habits.

We evaluated a total of 240 patients with symptoms and sign of UTI. Out of this, 214 (89.17%) yielded significant bacteriuria (Table **2**). *E. coli* (28.5%) and *Staphylococcus aureus* (28.0%) were the most common isolates in the cases of UTI in the study. However, there was no significant difference (p > 0.05) in the rate of occurrence of all the isolates (*E. coli, S. aureus, Salmonella* spp and *P. aeruginosa*).

From the antibiogram study, (Table **3**) it can be observed that the carbapenems, monobactam, macrolide and fluoroquinolones (Meropenem, Aztreonam, Azithromycin and Levofloxacin respectively) performed better than the rest of the antibiotics tested with the fluoroquinolone proving to be the most ideal drug in the management of bacteria UTI in the study centre. It was observed that the *Escherichia coli* isolates responded worst to the commercial antibiotics tested (mean % susceptibility = 22.05%). A Two-Way ANOVA of the susceptibility of the isolates to the drugs shows that the kind of antibiotic used extremely affected the microbial susceptibility and accounted for 79.87% of the total variance with a p value of < 0.0001. It is therefore inferred that the antibiotic type significantly affected the microbial susceptibility. Also, the kind of bacterial isolate accounted for only 6.00% of the total variance with a p value = 0.0211. This also showed that the isolate type significantly affected the effects of the drugs.

Bonferroni post-tests analysis showed that there was a significant difference in the effect of Meropenem on the *Salmonella* spp and *P. aeruginosa* isolates (p <0.01) and on *S. aureus* isolates (p < 0.05) compared with the effects of Ceftazidime. Also, effects of Aztreonam on the *Salmonella* spp and *P. aeruginosa* isolates compared with the effects of Ceftazidime (p <0.01). A comparison of Azithromycin and Ceftazidime showed a significant difference for the *Salmonella* spp and *P. aeruginosa* (p <0.01) and for *S. aureus* (p < 0.05) isolates. Same effects were observed between Meropenem and Cefoxitin. Aztreonam was significantly better than Cefoxitin on the *Salmonella* spp and *P. aeruginosa* isolates (p < 0.01). Azithromycin was significantly better than Cefoxitin against *Salmonella* spp and *P. aeruginosa* isolates (p < 0.05) and against *S. aureus* isolates (p<0.05). Same was also true of Azithromycin compared to Fosfomycin. Meropenem was significantly better than Fosfomycin against *Salmonella* spp and *P. aeruginosa* isolates (p < 0.01) and against *S. aureus* isolates (p <0.05). Aztreonam was significantly better than Fosfomycin against *Salmonella* spp and *P. aeruginosa* isolates (p < 0.01). Same was also true of Meropenem and Azithromycin compared to Cefuroxime. Meropenem and Aztreonam were significantly better than Ceftriaxone against *P. aeruginosa* isolates (p < 0.05). Only Levofloxacin showed a significantly better effect on all the isolates (*Salmonella* spp, *P. aeruginosa*, *Escherichia coli* and *S. aureus*) p < 0.05 compared to most drugs used. *Escherichia coli* (especially) and *S. aureus* isolates showed significantly higher resistance to several of the commercial antibiotics (p >0.05).

## 4 DISCUSSION

The study centre is a state owned tertiary hospital located in Awka, the state capital and is engaged in research and training of medical and nursing students and specialist doctors. The state capital, Awka, is surrounded by several rural settlements and this could be the reason for the higher patronage by the rural communities. Many of these communities do not have standard health facility and so the patients are seen flooding the available facility nearest to them. The dearth of these health facilities is one of the reported reasons why the existing ones are always over-burdened and facilities easily break down [16]. Our study also showed that the incidence of UTI is highest among the middle age group (22-36 years). This is understandable because this age group covers the very sexually active and prime reproductive years of a woman. Sexual activity and pregnancy were reported to increase the risk of having UTI [5, 9]. The incidence of bacterial UTI was found to be higher (though not significant, p value < 0.05) with decreasing level of formal education. This may be associated with socio-economic status and orientations of the study participants. It had been documented that formal education enhances socio-economic status and health of an individual such as orientations towards personal hygiene [17, 18]. The unmarried females were observed to have higher incidence compared to their married counterparts probably because the greater percentage of them were sexually active and in their explorative ages of sexual drive [19].

HIV infection and pregnancy both lower the immune status of an individual and so significantly enhance the incidence of UTI as observed in our study. Pregnancy, additionally, causes hormonal and mechanical changes that increase the risk of urinary stasis and backward flow of urine from the bladder into the ureters/kidneys. Pregnancy also presents additional challenge to the genital hygiene because of high incidence of leucorrhea gravidarum. All these factors combine to increase the incidence of UTI in pregnancy. Similar observation had been reported by other researchers [20, 21].

*Escherichia coli*, an *Enterobacteriaceae*, is the major aerobic organism residing in the intestine and is the most commonly reported cause of UTI [22, 23] being a common faecal contaminant. Due to the short urethra of females and closeness of the female anus and the vagina to the urethra, the organism is most likely to be inoculated into the urethra during the process of anal cleaning after defecation and during sexual intercourse. The predominance of *Escherichia coli* in cases of UTI is supported by our finding and that of other researchers [24, 25]. It is also not surprising that *Staphylococcus aureus* is implicated in UTI in this study knowing that they are normal skin microbiota. They can be easily inoculated into the urethra from the surrounding skin during anal cleaning after defecation. Similar result had been reported [26]. The other bacteria *Salmonella* spp and *Pseudomonas aeruginosa* had also been reported to cause UTI [26-28].

Urinary Tract Infection is a common contagion in humans. The distribution and antibiogram of the uropathogens is constantly changing due to clinical failure resulting from misuse or overuse of the commonly available antibiotics. Increasing antimicrobial resistance among uropathogens greatly increase the economic burden of UTI treatment. From the antibiogram study, (Table **3**) it was observed that the isolates were resistant to several commercially available antibiotics with resistance mostly seen against the cephalosporins. It was also observed that the beta-lactamase inhibitor, Ceftriaxone/Sulbactam, performed poorly as well as Fosfomycin – a broad-spectrum naturally occurring antibiotic obtained from several *Streptomyces* and *Pseudomonas* species. Fosfomycin is indicated in the treatment of acute uncomplicated lower urinary tract infections and is usually administered as a single oral mega-dose. Several authors had also observed similar resistance to Fosfomycin and offered that the possible mechanisms of resistance to the drug include: reduced permeability to the antibiotic, modification of the antibiotic’s target enzyme –MurA, inactivation of the antibiotic *via* the production of fosfomycin resistance proteins by the pathogenic bacteria and finally modification of the fosfomycin binding site Cys115 [29, 30]. Meropenem, aztreonam, azithromycin and especially levofloxacin proved to be the most promising drugs in the management of UTI in the study centre. This study also revealed a wide range of susceptibility of the isolates to the tested antibiotics. The need for organism-specifically susceptible drugs in the management of UTI is therefore emphasized in this study.

The incidence of resistant *Escherichia coli* in the study center has been reported by other researchers [31, 32] who demonstrated the presence of ESBL producing *Escherichia coli*.

## 5 STRENGTHS AND LIMITATIONS OF THE STUDY

Hitherto, clinicians had relied on the information on drug detailing as given by drug companies’ sales representatives for the treatment of UTI in the study centre and not on empirical evidence of susceptibility of the isolates. Also, epidemiological data on the microbial isolates in cases of UTI has been lacking. This study, therefore, provides a first-hand information or documentation on both scientific evidence of microbial susceptibility of the isolates as well as the microbial etiology of UTI in the study centre. This study is of international importance knowing that the world is a global village where people can migrate from one country to another with the attendant possibility of spreading resistant strains of microbes. A knowledge of the susceptibility profile as offered by this study will help clinicians offer objective treatment of UTI and so reduce intercontinental spread of resistant microbial strain.

However, the study did not provide the molecular bases of resistance to the commercial antibiotics. It also did not establish the presence of extended beta-lactamase producing isolates as a possible cause of resistance to the antibiotics.

## CONCLUSION

In this study, *E. coli* and *S. aureus* were the predominant pathogens. The bacterial isolates were resistant to the commonly prescribed drugs and so left the clinicians with only few alternative drugs for UTIs treatment. The pattern of microbial resistance to antibiotics suggests that Ceftazidime, Fosfomycin and Cefoxitin may not be the appropriate first-line agents in the empirical treatment of UTIs, instead; Levofloxacin, Meropenem or Aztreonam may be considered. More studies on this topic will substantiate this finding. Routine surveillance and monitoring studies need to be constantly conducted to update clinicians on the prevalent pathogens and the rational and empirical treatment of UTIs. Aggressive and consistent health education using all possible social media is also recommended to combat the menace of drug resistance occasioned by inappropriate antibiotic use.

## Figures and Tables

**Table 1:** Characteristics of study participants (N = 240).

**Variable**	**Number of Subjects Tested**	**Number (%) with Significant Bacteriuria**	**Chi-Square (X^2^) p Value**	**Correlation (% Bacteriuria) P Value**
**Location (Residence)**			p value = 0.0691	
Urban	40	32 (80.00)		0.0962
Sub-Urban	85	75 (88.24)		
Rural	115	107 (93.04)		
Age Group (Years)*				
12 – 16	19	16 (84.21)	**ANOVA p value < 0.0001**	0.1674
17 – 21	20	17 (85.00)		
22 – 26	54	47 (87.04)		
27 -31	50	49 (98.00)		
32 - 36	65	60 (92.31)		
37 – 41	20	17 (85.00)		
42 - 46	4	2 (50.00)		
47 - 51	3	2 (66.67)		
52 – 56	5	4 (80.00)		
**Highest Education Completed**				
No formal education	31	29 (93.55)	p value = 0.0816	0.0517
First School leaving	52	48 (92.31)		
Senior Secondary Certificate (or equivalent)	84	77 (91.67)		
Tertiary Education	51	44 (86.27)		
Higher degree	22	16 (72.73)		
Marital Status*				
Single	125	118 (94.40)	p value < 0.0001	0.9192
Widowed	15	9 (60.00)		
Divorced	12	7 (58.33)		
Still Married	88	80 (90.91)		

**Table 2:** Frequency of occurrence of the Isolates.

**Organism**	**Number of Isolates**	**Incidence Rate (%)**	HIV Status*		Patients Age (yrs.)*									Pregnancy Status*	
			**Positive (n = 120)**	**Negative (n = 120)**	**12 - 16**	**17 - 21**	**22 - 26**	**27 - 31**	**32 -36**	**37 - 41**	**42 - 46**	**47 - 51**	**52 - 57**	**Positive (n = 120)**	**Negative (n = 120)**
***E.coli***	61	28.5	46	15	3	4	15	16	14	5	0	1	3	45	16
***S. aureus***	60	28.0	31	29	6	4	14	15	16	5	0	0	0	33	27
***Salmonella* spp**	49	22.8	36	13	3	5	9	11	15	4	1	1	0	38	11
***P. aeruginosa***	44	20.5	32	12	4	4	9	7	15	3	1	0	1	34	10
**TOTAL**	**214**	**100**	**145**	**69**	**16**	**17**	**47**	**49**	**60**	**17**	**2**	**2**	**4**	**136**	**78**
			**X^2^ p value = 0.0188**			**ANOVA p value < 0.0001**								**X^2^ p value = 0.0256**	

**Table 3:** Drug Susceptibility profile of Isolates.

**Bacterial Isolate**	**% Susceptibility**										**Mean% Susceptibility**	**ANOVA**
	**CAZ**	**FOX**	**FOS**	**CXM**	**CEF**	**C/S**	**MEM**	**ATM**	**HVX**	**LVD**		
***Salmonella* species**	0.0	0.0	3.3	5.0	23.0	39.3	74	78.7	49.2	86.9	35.94	**p value = 0.0211**
***P. aeruginosa***	0.0	0.0	6.1	14.3	18.4	37.0	86	71.4	61.2	81.6	37.60	
***Escherichia coli***	0.0	13.3	1.6	3.3	8.3	17.0	37	75.0	5.0	60.0	22.05	
***Staphylococcus aureus***	0.0	0.0	0.0	14.0	23.0	43.2	59.1	59.1	73.0	90.9	36.23	
**Mean % Susceptibility**	0.00	0.00	2.75	9.15	18.18	34.13	64.03	71.05	47.1	79.85		
**ANOVA**	**p value < 0.0001**											

**Key:** CAZ = Ceftazidime, FOX = Cefoxitin, FOS = Fosfomycin, CXM = Cefuroxime, CEF = Ceftriaxone, C/S = Ceftriaxone/Sulbactam, MEM = Meropenem, ATM = Aztreonam, HVX = Azithromycin, LVD = Levofloxacin
